# Adapting the Gamified Educational Networking Online Learning Management System to Test a Decentralized Simulation-Based Education Model to Instruct Paramedics-in-Training on the Emergency Intraosseous Access and Infusion Skill

**DOI:** 10.7759/cureus.55493

**Published:** 2024-03-04

**Authors:** Amanpreet K Jolly, Dilothi Selvarajah, Julia Micallef, Andrei Torres, Dale Button

**Affiliations:** 1 Biological Sciences, Ontario Tech University, Oshawa, CAN; 2 Health Sciences, Ontario Tech University, Oshawa, CAN; 3 Computer Science, Ontario Tech University, Oshawa, CAN; 4 Paramedicine, Durham College, Oshawa, CAN

**Keywords:** healthcare simulation, emergency medical service, paramedic training, decentralized simulation-based education, intraosseous access

## Abstract

Intraosseous (IO) access and infusion is a safe and rapid alternative to intravenous access in obtaining vascular access for administering fluids and drugs. Healthcare professionals, such as primary and advanced care paramedics, use IO access and infusion in emergency circumstances where peripheral intravenous routes are inaccessible. IO access skills require hands-on training, which can be done remotely if the participants have access to simulation, instructions, guidance, and feedback. For the purpose of moving the training outside of the simulation laboratories, we have developed (1) an inexpensive and scalable three-dimensional (3D) printed and silicone-based advanced adult proximal tibial IO access and infusion simulator and (2) a unique learning management system (LMS) for remote simulation-based training. The LMS was built using the Django platform and supports experiential learning by providing access to educational and instructional content (including virtual simulation and serious games), allowing peers to communicate among themselves and with subject-matter experts, provide and receive feedback asynchronously, and engage in learning using gamification elements. The aim of this technical report is to describe the process of development and the final product of the LMS as a research and educational tool to scaffold remote learning of emergency IO skills by paramedics-in-training.

## Introduction

As we move away from the stringent restrictions of the coronavirus disease 2019 (COVID-19) pandemic, the North American healthcare system now faces a human resource crisis that is challenging education similar to the pandemic [1]. The primary emphasis on patient care by healthcare workers due to staff shortages hinders their ability to adequately fulfill educational responsibilities [1]. Traditionally, the physical presence of front-line staff with respect to in-patient and out-patient settings in health professions education has served as an integral part of early clinical experience [2]. These limitations, coupled with the logistical difficulties and high costs of centralized simulation-based education (Ce-SBE), a model in which students practice skills in simulation laboratories, sparked a growing interest in another form of simulation to continue the training of healthcare providers [3]. The high costs of the Ce-SBE model often derive from the number of instructors required at the teaching sites and their respective compensations, transportation costs for participants, and more [3]. As we move increasingly towards online education, where it can be as effective as traditional in-person training, Ce-SBE hinders the feasibility of the current healthcare system. The challenges posed by COVID-19, including issues like social distancing and limited resources, further support the practicality of moving away from this approach. As a result, the focus shifted away from Ce-SBE towards decentralized simulation-based education (De-SBE), a model in which students practice skills outside of laboratories, to understand if and how De-SBE can function to augment the more traditional use of simulation [3]. De-SBE functions by utilizing simulators as training tools, allowing learners to engage in training from their own homes [3]. This approach is reinforced by the integration of a learning management system that delivers instructions, guidance, and feedback creating a convenient environment for the learners [3].

The development of a De-SBE model could potentially lead to continuous, undisrupted training and lower training costs and provide students with a unique chance to tailor their learning to match their own needs and pace, which is a concept known as mastery-based learning [4]. This concept can be used as an educational approach that allows learners to develop skills through deliberate practice resulting in very high levels of performance outcomes with little variation in outcomes among learners [4]. Mastery-based learning functions by deconstructing tasks into a series of smaller and increasingly complex steps [4]. The learner progresses through these steps by mastering each one before advancing to the next, applying the skills acquired in the preceding steps [4]. To fulfill the aforementioned voids created by inaccessible on-site learning circumstances, the switch from Ce-SBE to De-SBE may be feasible.

In cases where patients undergo shock, severe dehydration, cardiac arrest, and major trauma or encounter airway compromise, intraosseous (IO) administration serves as an alternative for delivering fluids and medication [5]. IO access and infusion provides a safe and rapid alternative to intravenous access for obtaining vascular access [5]. Healthcare professionals, including paramedics, employ IO access and infusion in emergency circumstances where peripheral intravenous routes are unavailable [5]. The typical method of teaching the IO skill is commonly conducted in a classroom environment with an instructor guiding students through each step [5]. To improve comprehension, this structured approach combines hands-on practice supplemented by multimedia resources and interactive discussions [5].

​​For the purpose of moving the training outside of the simulation laboratories (i.e., converting the training into a De-SBE model), we have developed an inexpensive and scalable three-dimensional (3D) printed and silicone-based advanced adult proximal tibial IO access and infusion simulator [6] and a learning management system (LMS) for the simulation-based acquisition of psychomotor skills [7] that can be adapted to structure training. 

The aim of this technical report is to describe the process of development and the final product of an online LMS to scaffold remote learning of the emergency IO skills by advanced care paramedics (ACPs) in training, henceforth referred to as the participants. The adopted LMS was the Gamified Educational Network (GEN) using the Django platform. GEN was developed to support experiential learning by providing access to educational and instructional content (including virtual simulation and serious games). In addition, GEN allows peers to communicate among themselves and with subject-matter experts (SMEs), provide and receive feedback asynchronously, and engage in learning using gamification elements [7], henceforth referred to as the Gamified Educational Network Intraosseous (GEN IO). Learner's feedback was measured through system usability survey (SUS) scores and was used to explore the educational and research implications of GEN IO [8].

## Technical report

Context

Framework

IO access and infusion is a secure and fast substitute for intravenous access when it comes to obtaining vascular access for delivering fluids and medications [5]. Medical personnel, including paramedics, rely on IO access and infusion during emergency situations when peripheral intravenous routes are not available [5]. The intended learners are college-level students in their one-year graduate certificate program for advanced care paramedics at Durham College (Oshawa, Ontario) [9].

The GEN IO can be used as a research tool as it encompasses pretest, practice, and posttest elements to measure the efficacy of the model. Within the pretest section, participants are to attempt the IO skill once and upload a video of themselves demonstrating this skill as a baseline measure. The participants are to attempt the IO skill as many times as necessary until they achieve a sense of proficiency and confidence. The posttest section allows the participants to attempt the IO skill for a final time and upload a video recording of this attempt. The purpose of this section is to capture the last attempt of the IO skill by the participant to compare it to the baseline measure, which is the pretest attempt. Once participants complete the IO skill training, they are given the chance to assess their own performance in the "Self-Assessment" section. The self-assessment data allows researchers to gather information on participants' perceptions of their posttest performance.

**Figure 1:**
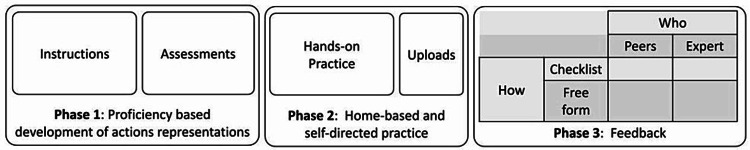
Model illustrating three phases of learning and the components adopted for the GEN IO. GEN IO: Gamified Educational Network Intraosseous

GEN IO was developed as an experimental LMS to test what unique features need to be implemented in an LMS that supports the De-SBE model of training. It was initially designed based on the conceptual framework proposed by Dubrowski et al. [10]. The framework shown in Figure 1 focuses on remote and online psychomotor skills development that consists of four key components of the LMS which correspond to the four parts of deliberate practice: (a) opportunities for hands-on repetition, mechanisms to increase (b) motivation and (c) consistency in LMS use, and (d) accurate feedback [10]. 

Beginning with Phase 1, active instructions are provided to students with error-free instructional videos [10]. Following this logic, instructional videos of error-free IO skill performance were integrated into GEN IO using advanced and simple IO models and equipment. The participants were permitted to review the video as often as necessary until they were comfortable with the skill. 

Next, in Phase 2, participants engage in self-directed practice where they use the simulators developed for the skill to practice in their setting; if more instruction is needed, they may return to the instructional materials [10]. Once they feel satisfied with their performance, they will record themselves doing a test trial and upload it onto the LMS for review [10]. In GEN IO, participants are required to attempt the IO skill once in a "Pretest" and provide a video recording of themselves doing so. The objective of this section is to record the participant's initial attempt at the IO skill as a baseline measure. The next section of GEN IO is "Practice," which allows participants to attempt the IO skill as many times as they would like without recording themselves. The following section is the "Posttest," where participants are asked to attempt the IO skill one final time and upload a video recording of themselves doing so. A "Retention Test" is also included, in which participants are requested to return after a week of not practicing to attempt the IO skill again using only their memory and once again upload a video of themselves performing this trial. We ensured that participants have refrained from practicing the IO skill through verbal verification on the day of their retention test; we relied on their honesty in this matter. 

Lastly, in Phase 3, the learners will either get feedback from their peers, an expert, or a combination of both on their test trial [10]. Feedback can be given in the form of comments or using a checklist, such as an Objective Structured Assessment of Technical Skills (OSATS) [10]. Similarly, in GEN IO, the test trials were marked by a paramedic instructor (DB). 

Educational Setting 

The educational context chosen was Durham College (Oshawa, Ontario). An ethics approval was obtained for this work by the Durham College Research Ethics Board (approval number: 241-2122). Durham College has a paramedic program for ACPs [9]. One problem that this local population faced was that they completed all of their training including the IO skill in their simulation lab (Ce-SBE model) and they did it based on the number of hours, not competencies that are reached by the learners. To move training outside of their simulation lab and transform it into a De-SBE model while integrating a competency-based approach, we made two models. These models are called the simple IO model and the advanced IO model. The simple IO model is a bone with a cut made from the upper leg: from the knee to the middle of the shin [11]. A small box hole was created on the bottom for sliding the bone onto a slide to be clamped [11]. The simple IO simulator was made with Fusion360 (Autodesk Inc., San Rafael, CA) [11]. Using Ultimaker Cura (Ultimaker B.V., Utrecht, The Netherlands), the digital design was adjusted to have a 3 mm wall thickness for better drill resistance and a 30% infill in a line pattern to mimic bone marrow in the tibia [11]. Then the design underwent printing using white Ecotough polylactic acid filament (Mississauga, ON) on an Ultimaker S5 3D printer (Ultimaker B.V., Utrecht, The Netherlands) [11]. The advanced IO model was 3D-printed, with silicone skin and a sliding bone for IO access [11]. To create the advanced IO simulator, the leg's dimensions and shape were derived from a human leg, which underwent 3D scanning using an Artec Space Spider scanner (Luxembourg, Europe) [11]. It was customized to 75% of its original size for a more manageable prototype [11]. In SolidWorks, the scanned leg was modified to have a cut from the knee to the middle of the shin, creating a small box for the bones to slide in [11]. Finally, the leg was printed utilizing skin-tone polylactic acid (PLA) filament on an Ultimaker S5 3D printer [11].

Along with this, there are two modules in the GEN IO for each model to support ACPs' learning on the simple IO model and ACPs' learning on the advanced IO model. Different versions of the IO modules are designed to match the skill levels of beginners, intermediates, and experts. For the purposes of this technical report, we will describe the development of the module relevant to the advanced model only which will be henceforth referred to as the GEN IO Module.

This paper will describe how we went about (1) setting up the GEN IO and (2) obtaining user experience data to inform how well the GEN IO was perceived by the ACPs at Durham College. 

Inputs and design process

Team

The research team included a researcher well-versed in Computer Science (AT), seven researchers in Health Sciences consisting of both master's and undergraduate students (MS, SA, JM, AJ, DS, MP, and WF), and a simulation-based medical education expert (AD). In addition, one paramedic/instructor (DB) was part of the research team to ensure that the GEN IO Module represented training needs. 

*Participants*****

There were 11 (n=11) participants consisting of ACPs in training at Durham College. 

GEN IO

The GEN IO platform was developed using the Django platform which is a high-level Python web framework known for its emphasis on rapid development and clean design principles [12]. By utilizing Django, developers were able to create a versatile platform for experiential learning, which offered access to educational content, virtual simulations, and serious games [12]. Additionally, the GEN IO facilitates communication among peers and SMEs, enabling asynchronous feedback, and incorporates gamification elements to enhance the learning experience [7]. 

GEN IO adheres to the challenge point framework (CPF). CPF derives from research involving both laboratory and field studies that have consistently demonstrated improvements in learning outcomes [13]. It serves as a tool for representing and uncovering insights about the connections between practice strategies and the resulting learning outcomes [13]. This framework explains the influence of specific practice approaches on an individual's learning process [13]. The main concept highlights the need to effectively engage both the cognitive and physical systems to enhance optimal learning [13]. Importantly, it draws attention to the move from short-term practice to long-term learning in order to foster highly efficient and stress-resistant learning [13]. The GEN IO has four different variations. The IO1 Module is designed to assist ACPs in learning the fundamentals of the simple IO model. The IO3 Module is designed to assist ACPs in learning the fundamentals of the advanced IO model. The other two modules are for more complex versions of the simulator and for learners of different skill levels. Therefore, the GEN IO as a whole supports the idea of adaptive learning built on the principles of optimal CPF [13]. 

Instructional Materials

The GEN IO was observed in person during the testing with participants. The GEN IO first orients the participant into the training by outlining the overall objective: to teach and advance the participant in the IO skill following the information in the module. Next, the participant is presented with an instructional video demoing the IO skill with specific IO simulators and medical equipment. The IO model is intended to be 3D-printed by the participant if they are attempting to acquire the IO skill remotely following De-SBE, using a link provided to the digital design of the IO model, which will also be made publicly available on an online resource-sharing platform (i.e., GitHub). They can also be purchased or requested from our lab by connecting with us via maxsimhealth.com. 

The specific IO medical equipment is also the participant's responsibility to obtain/purchase for the training. In the case of using GEN IO, all relevant materials were provided to the participants, and they were simply accessing the LMS component of the De-SBE. In the module, participants will also have access to additional informational resources on the IO skill, such as PowerPoint slides. Once the participant views these materials, they are instructed to practice the IO skill using the specific IO model and medical equipment, as demonstrated in the instructional video, until they feel proficient. When the participant feels comfortable with the IO skill, they are requested in the module to attempt the IO skill one last time while video recording themselves. The participant is instructed to upload the video recording in the module for instructor assessment.

*Process*****

**Figure 2:**
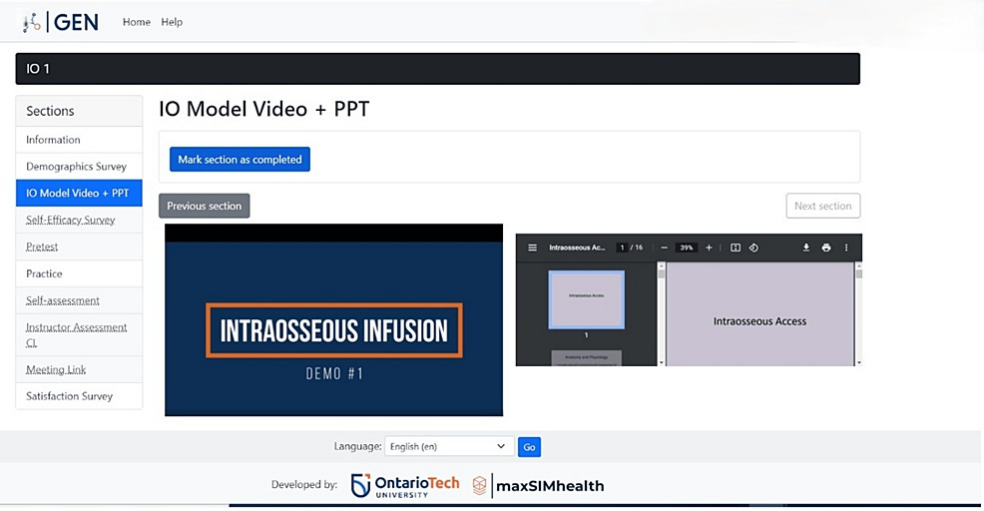
Educational component with video and PowerPoint on learning the GEN IO1 Module.

**Figure 3:**
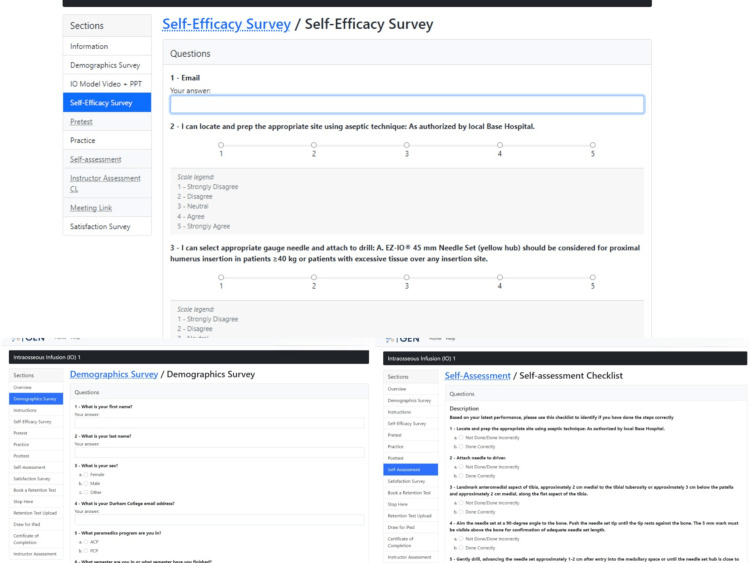
Research component with tests and surveys on the GEN IO1 Module.

Phase 1: prototype 1. The first phase focused on developing ideas for the product that would teach participants to learn the IO skill step-by-step remotely. The precise elements of GEN IO that were required were identified. The elements were divided into two components: educational and research. The educational component involved instructions, videos, and PowerPoint presentations on learning the IO skill, which is shown in Figure 2. The research component involved tests and surveys, which are shown in Figure 3. The research component was needed as the data sources helped to make design decisions. The types of tests and surveys used were a self-efficacy survey, pretest, posttest, practice, self-assessment, demographics, and satisfaction survey. 

Phase 2: prototype 2. With each component, various changes were made, including the renaming of the section (transfer test to retention test) and the deletion and addition of survey questions. Next, the instructor tested the GEN IO Module for its functionality ensuring that all the integrated components worked from the participants' perspective. This process evaluated whether their progress was saved, survey responses were saved, and the upload of any required files functioned. Some modifications were made to address the concerns that developed during this procedure. After numerous test runs and revisions, a well-developed product was created and integrated into the study for participant usage.

The development process of the GEN IO Module involved 11 revisions. First, an initial meeting between the researchers (MS and AD) took place to discuss the various components of the GEN IO. Based on the information gathered from the meeting, a preliminary structure of the GEN IO was drafted in Google Docs. A focus group interview (MS, AD, SA, AT) was held to gather feedback primarily from the computer science perspective to understand the feasibility of the design proposed in the draft, and a primary version of the GEN IO was built. Next, the researchers (MS and AD) provided feedback on the primary version of the GEN IO, and a finalized version of the GEN IO was made. A paramedic instructor, DB, was consulted to determine the appropriate educational resources to include in the GEN IO. DB provided us with an Objective Structured Clinical Examination (OSCES) checklist to make the assessment surveys which were integrated into GEN IO [14]. DB was then video-recorded performing the IO access and infusion procedure on the simple and advanced IO models according to the step-by-step guide, which was uploaded to the GEN IO as another resource. Finally, a PowerPoint was shared with the research team and added to the GEN IO. The total process took four weeks, from the development of the general idea to the creation of the final product, the GEN IO. Feedback and the process of building GEN IO are shown in Table 1. 

**Table 1:** Suggestions and feedback for the development of the GEN IO. IO: intraosseous; AD: Adam Dubrowski; CL: cooperative learning; GEN IO: Gamified Educational Network Intraosseous

GEN IO structure	Feedback/suggestions
Information	Change "Information" to "Overview." For "Description" include "This module is designed to help you learn IO infusion skills using a three-dimensional printed simulator that will be provided to you at Ontario Tech University." Remove "Scoreboard." Remove "Leaderboard." Remove "Module Progress."
Demographics Survey	Add "What is your Durham College email address?" Add "What is your first name?" Add "What is your last name?" Add "What is your sex?" Options: M, F, Other. Make sure all questions are mandatory.
IO Model Video + PowerPoint	Change the section name to "Instructions."
Self-Efficacy Survey	Remove email textbox. Update survey. Make sure all questions are required.
Pretest	Include a description: Please attempt the IO skill once on the IO model. Record yourself while doing this attempt. Include an upload video feature (make it identical to the Practice section). Only proceed to the next section once the upload is complete.
Practice	We do not need the name or the description text box OPTIONAL. If participants record on their phone, can they upload from their phone to GEN IO on this section? YES. What is the max size video? 2 GB. Change the description to "Practice the skill until you feel proficient and keep a mental note of the number of times you attempt the skill." Video record your last attempt and upload it here. Add a "quiz question" or an "open field text box" asking "Please enter the number of times you attempted the skill." After you do both the upload video and the entry into the text box, then you can proceed to the next section.
Self-Assessment	Check with AD if we are going with this questionnaire above which is the CL survey or self-efficacy survey. Remove email. Make sure all questions are required, I was able to fill out a few and then I could successfully submit and move to the next section.
Instructor Assessment CL	Remove this section completely.
Meeting Link	Make sure to mark the section as completed since it is required before one can move to the next section. Remove the bullet point but keep the link. Remove extra spacing.
Satisfaction Survey	Make sure questions' responses are mandatory. Remove the problem-solving subheader that appears twice. Suggestion: include a completion page after the entire module is completed.
Book a Posttest	The test appointment link does not go to Google Forms.

Assessment

Assessment of GEN IO was done by participants where they completed a SUS survey hosted in GEN IO. SUS is a dependable, low-cost usability scale that is used for worldwide assessments of system usability [8]. Ten questions on a 5-point Likert scale addressed a number of factors of system usability, such as the need for support, training, and complexity (Table 2). The SUS yields a single number representing the overall usability [8], and although the SUS score ranges from 0 to 100, they are not percentages and should be considered in terms of their percentile ranking [15]. A SUS score above 68 is considered above average, and anything below 68 is below average [15].

**Table 2:** SUS questionnaire hosted on the GEN IO Modules. SUS: system usability survey; GEN IO: Gamified Educational Network Intraosseous

Question #	Question
1	I think that I would like to use this product frequently.
2	I found the product unnecessarily complex.
3	I thought the product was easy to use.
4	I think that I would need the support of a technical person to be able to use this product.
5	I found the various functions in the product were well integrated.
6	I thought there was too much inconsistency in the product.
7	I imagine that most people would learn to use this product very quickly.
8	I found the product very awkward to use.
9	I felt very confident using the product.
10	I needed to learn a lot of things before I could get going with this product.

Products and outcomes

Overview of the GEN IO Setup

**Figure 4:**
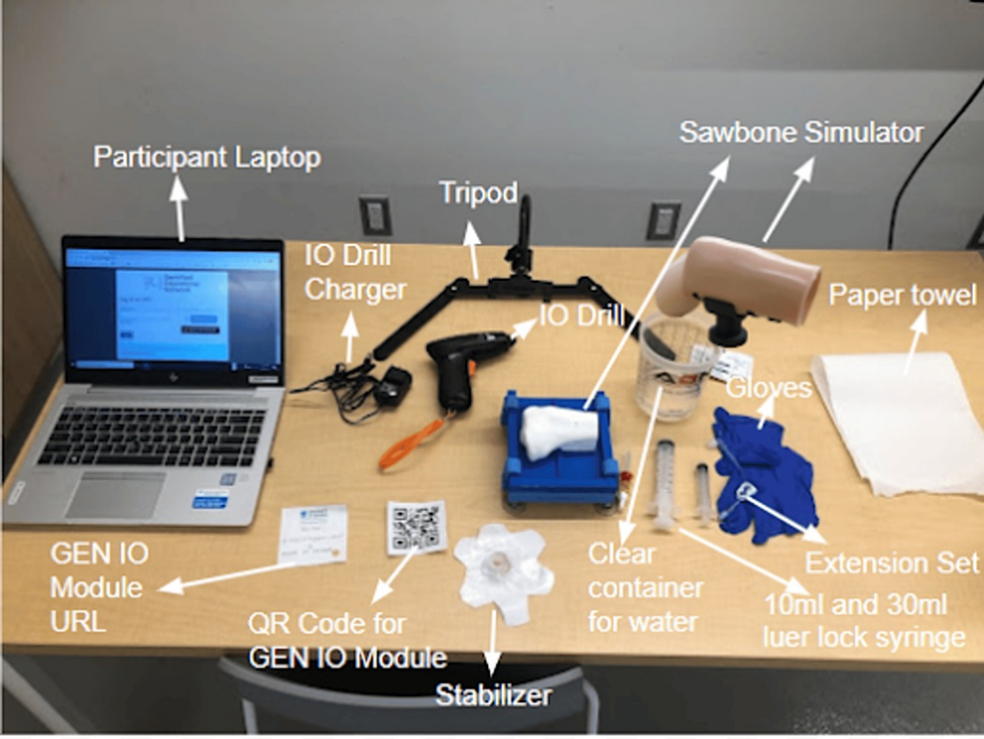
Physical setup of the GEN IO Module on a computer along with the simple IO, the advanced IO simulator, and the associated IO medical equipment. GEN IO: Gamified Educational Network Intraosseous; IO: intraosseous

The GEN IO is intended to be paired with the 3D-printed and silicone-based adult proximal tibia simulators and the associated medical equipment to train on the IO infusion and access skills (Figure 4). Below, both the educational and research aspects of GEN IO are presented. The educational aspect illustrates how GEN IO appears when integrated into a specific course for students. On the other hand, the research aspect showcases GEN IO during the research stages, where it serves as a source of data for design decisions. 

**Figure 5:**
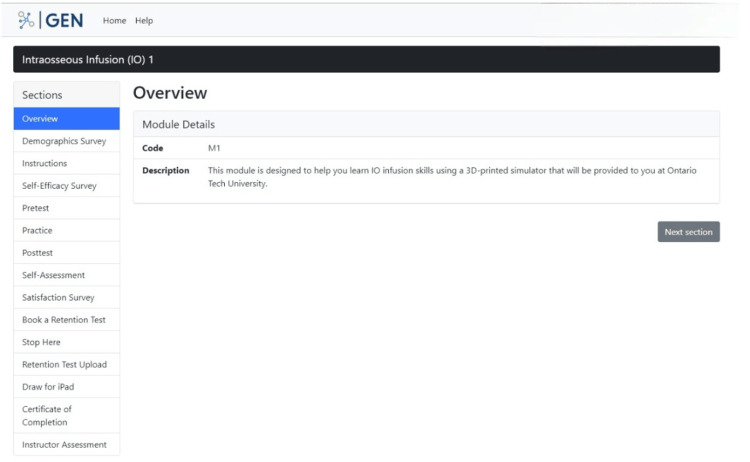
"Overview" section in the GEN IO1 Module.

Educational component:Once the participant logs into the GEN IO Module 1, they are first met with the "Overview" section that describes the purpose of the module and the relationship of the module to the study they are taking part in (Figure 5). This section orients the participants into the study. 

**Figure 6:**
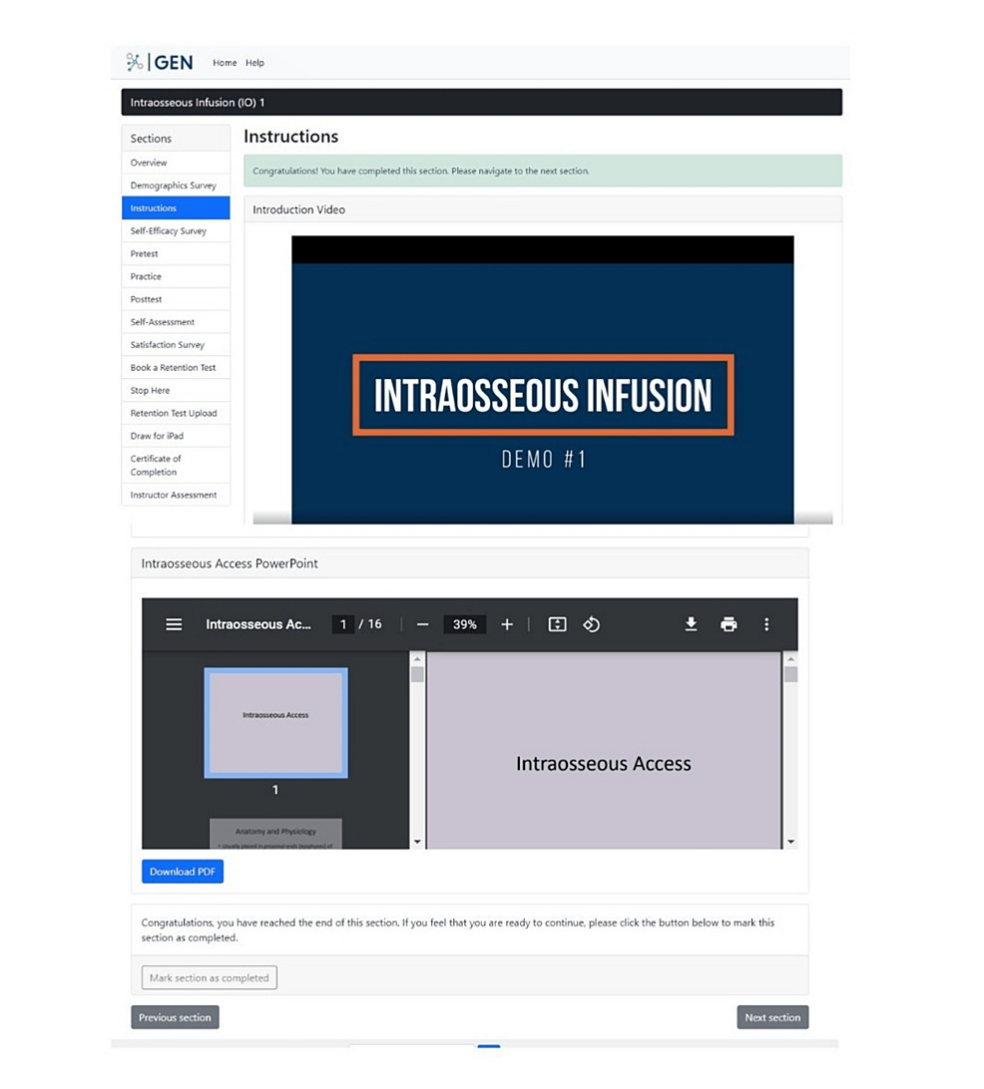
"Instructions" section in the GEN IO1 Module.

The participants can then access the "Instructions" section, where they can view a video showing a demo of the IO skill using the relevant IO model, equipment, and a PowerPoint presentation about the IO skill (Figure 6). 

**Figure 7:**
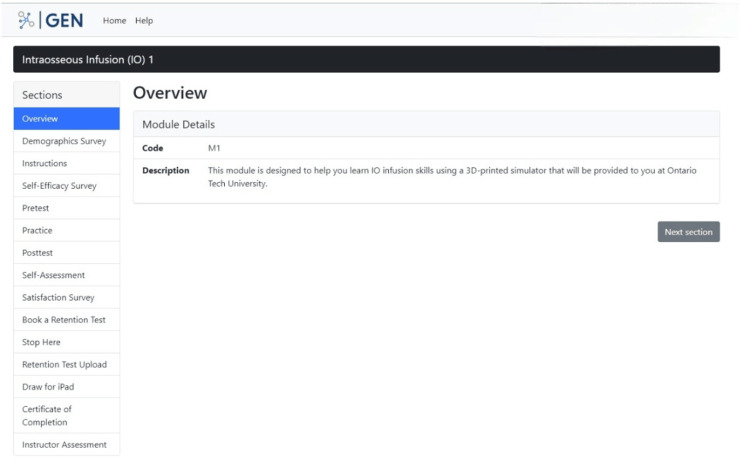
"Overview" section in the GEN IO1 Module.

Research component: Once the participant logs into the GEN IO Module 1, first, they are met with the "Overview" section that describes the purpose of the module and the relationship of the module to the study they are taking part in (Figure 7). This section orients the participants into the study. 

**Figure 8:**
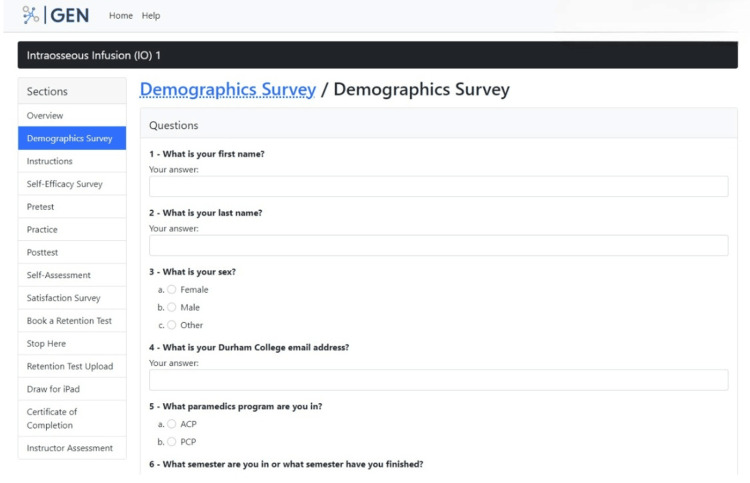
"Demographics Survey" section in the GEN IO1 Module.

Next, they move into the "Demographics Survey" section which is in a format that is a combination of multiple-choice and short-answer questions and asks the participant to enter their name, sex, program, experience in the IO skill, and other job-related training (Figure 8). This allows the research team to gather data on the participants taking part in the study. 

**Figure 9:**
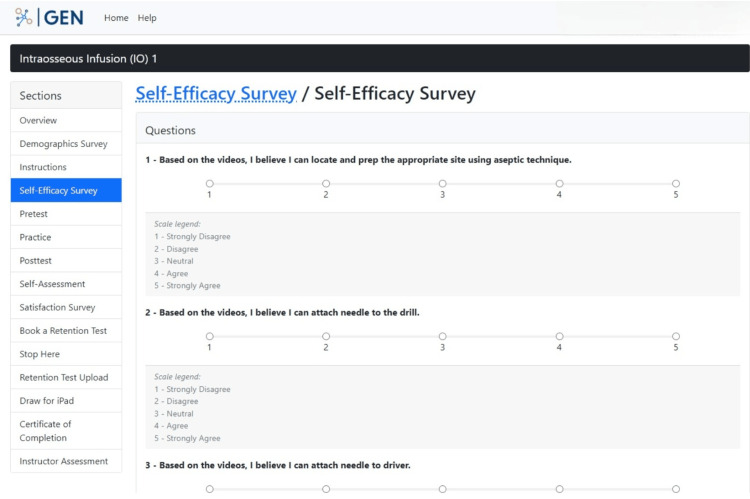
"Self-Efficacy Survey" section in the GEN IO1 Module.

A "Self-Efficacy Survey" section is made available to participants afterward to complete a survey on how confident they feel about performing the IO skill based on what they have learned from viewing the video and PowerPoint presentation in the previous section, "Instructions" (Figure 9). This section is intended to gauge how much participants believe in themselves prior to performing the skill for the first time.

**Figure 10:**
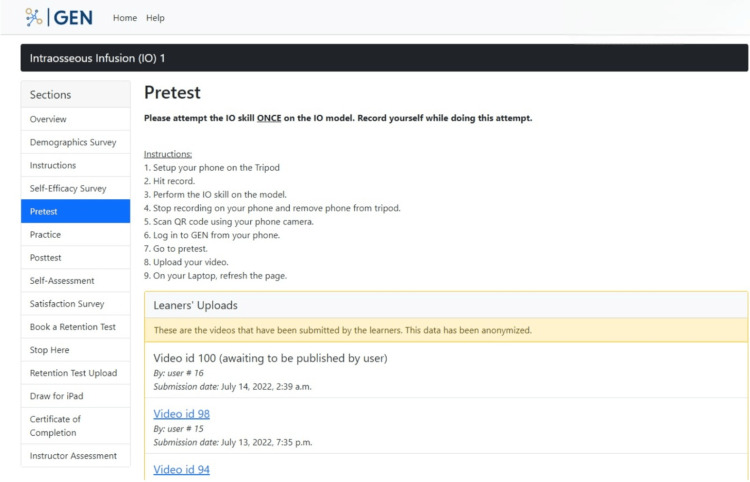
"Pretest" section in the GEN IO1 Module.

Participants are asked to attempt the IO skill once in the "Pretest" section and upload a video recording of themselves doing this attempt. Step-by-step instructions on how to record the attempt using the IO model and equipment, which is laid out in front of them during the study, are included in this section (Figure 10). The purpose of this section is to capture the first attempt of the IO skill by the participant as a baseline measure. This single attempt allows us to assess their current skill level. 

**Figure 11:**
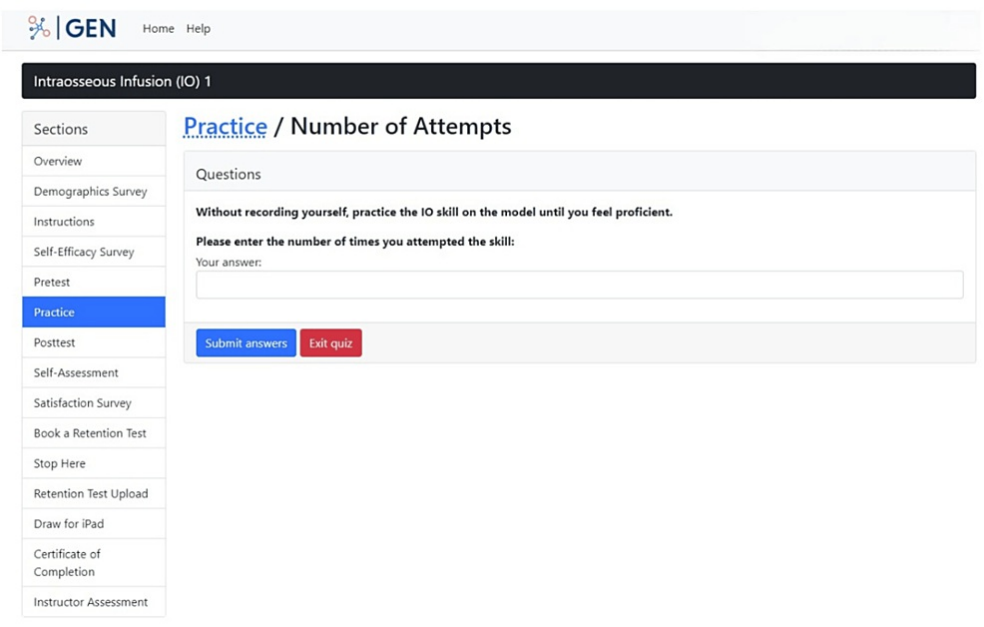
"Practice" section in the GEN IO1 Module.

The following section, "Practice," instructs participants to attempt the IO skill as many times as they would like until they feel proficient, without video recording themselves. Once they cease practice, in this section, participants are requested to enter the number of attempts in a textbox field (Figure 11). The purpose of this section is to obtain the number of attempts it takes participants on average to learn the IO skill. 

**Figure 12:**
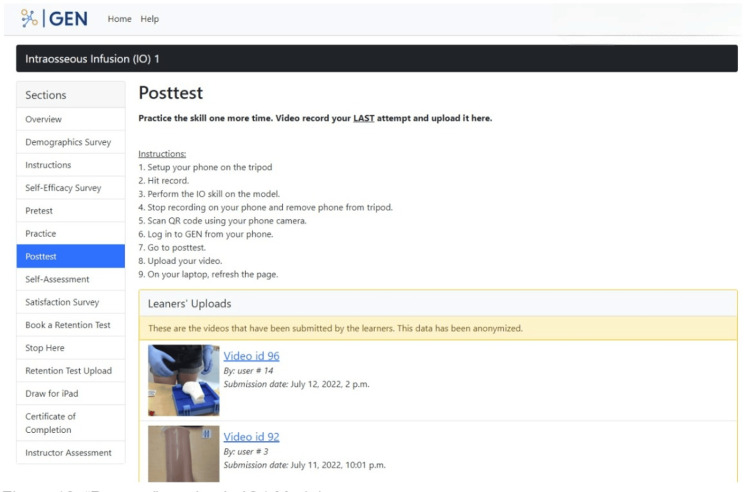
"Posttest" section in the GEN IO1 Module.

In the "Posttest" section, participants are required to attempt the IO skill one last time and upload a video recording of themselves doing this attempt. Step-by-step instructions on how to record the attempt using the IO model and equipment, which is laid out in front of them during the study, are included in this section (Figure 12). The purpose of this section is to capture the last attempt of the IO skill by the participant to compare it to the baseline measure, which is the pretest attempt. 

**Figure 13:**
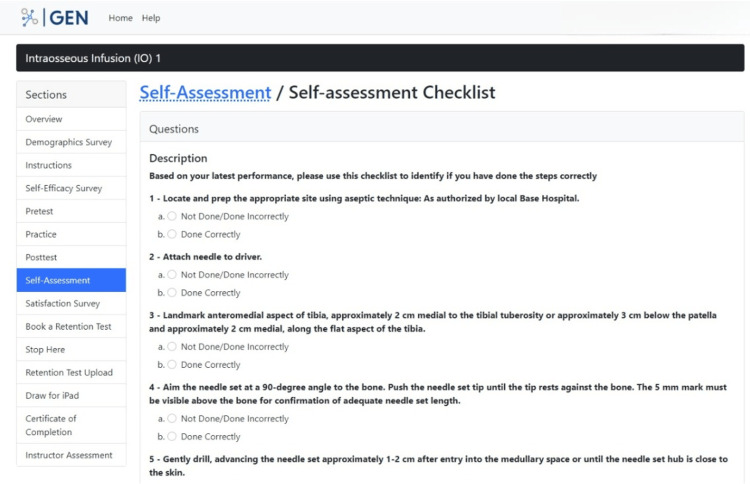
"Self-Assessment" section in the GEN IO1 Module.

After training on the IO skill, participants have an opportunity to rate themselves on their performance in the "Self-Assessment" section (Figure 13). This information provides the researchers with information on how well participants think they did in the posttest.

**Figure 14:**
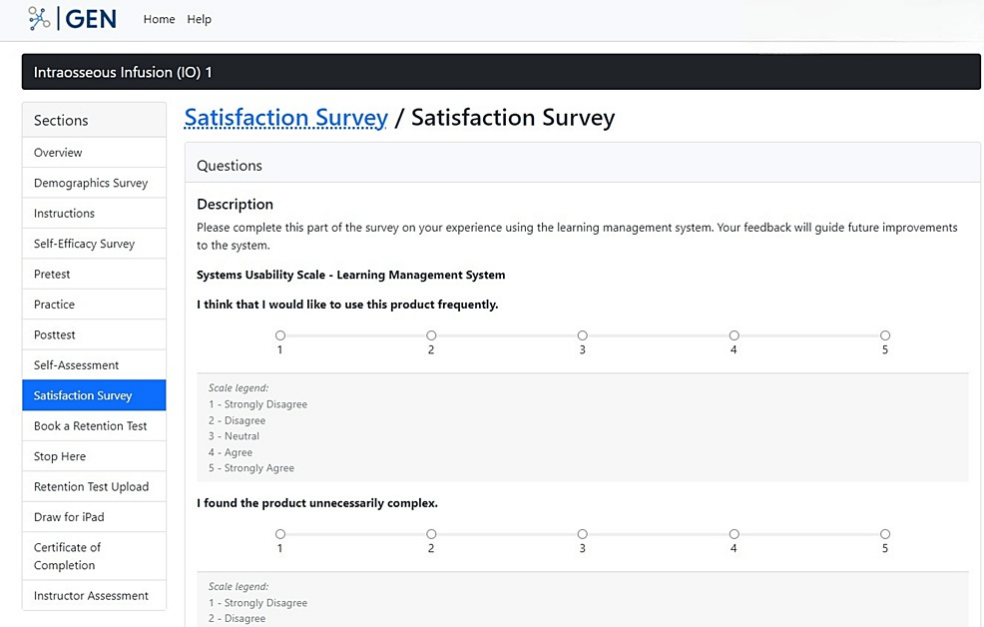
"Satisfaction Survey" section in the GEN IO1 Module.

A satisfaction survey, in the form of a quiz with multiple-choice and short-answer questions, in the next section offers participants a venue to share their thoughts and feelings on the overall study (Figure 14). This allows the researchers to receive feedback on the intervention in scope and possibly look to incorporate that in future iterations of the intervention.

**Figure 15:**
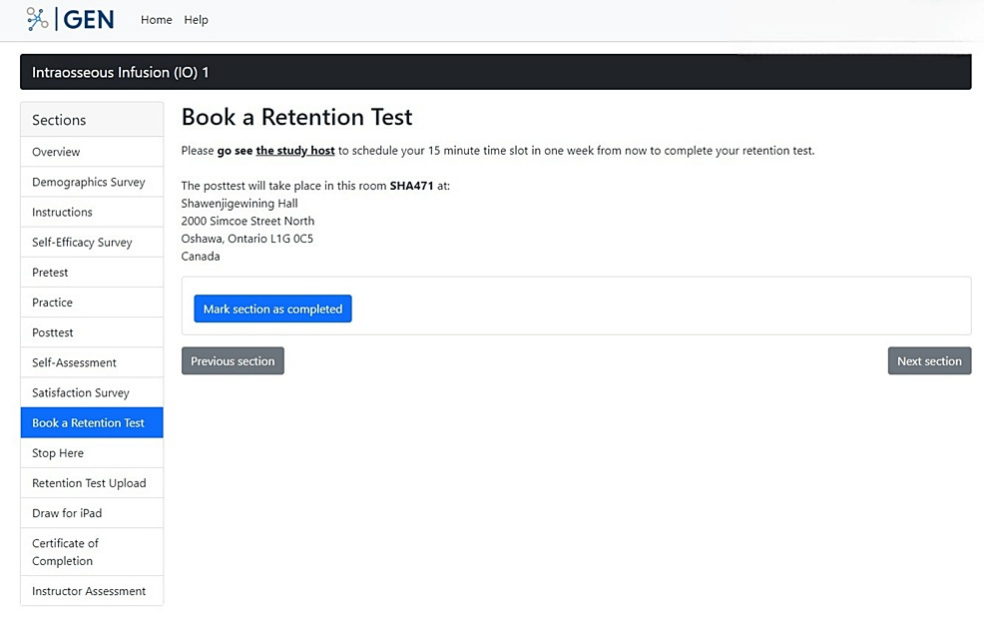
"Book a Retention Test" section in the GEN IO1 Module.

Participants are navigated to a section called "Book a Retention Test" that prompts the participants to stop practicing the IO skill for one week and to contact the study coordinator to schedule a retention test after this period (Figure 15). This is a way for researchers to schedule follow-up contact with the participants to understand how effective the training was through the intervention. 

**Figure 16:**
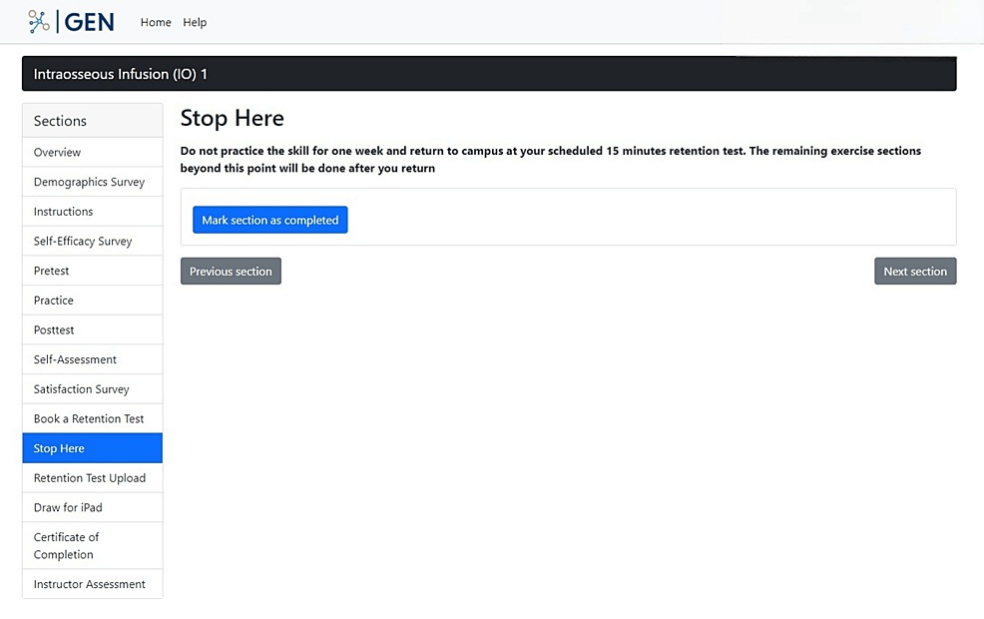
"Stop Here" section in the GEN IO1 Module.

Section "Stop Here" lets participants know that they have completed the first part of the study. It reminds them to not practice for a week and to show up for their scheduled retention test after one week (Figure 16). 

**Figure 17:**
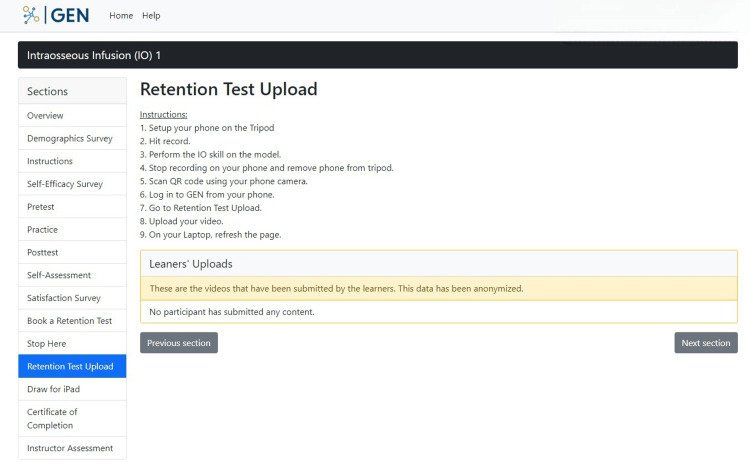
"Retention Test Upload" section in the GEN IO1 Module.

In the "Retention Test Upload" section, after one week of no practice, participants are asked to attempt the IO skill based on memory (without any reference to any resources in the module) and upload a video recording of themselves doing this attempt. Step-by-step instructions on how to record the attempt using the IO model and equipment that is laid out in front of them during the study are included in this section (Figure 17). The purpose of this section is to capture their attempt of the IO skill after one week and to see if any knowledge from previous practice of the IO skill, based on the instructions provided in the earlier sections of the GEN IO, was retained. 

**Figure 18:**
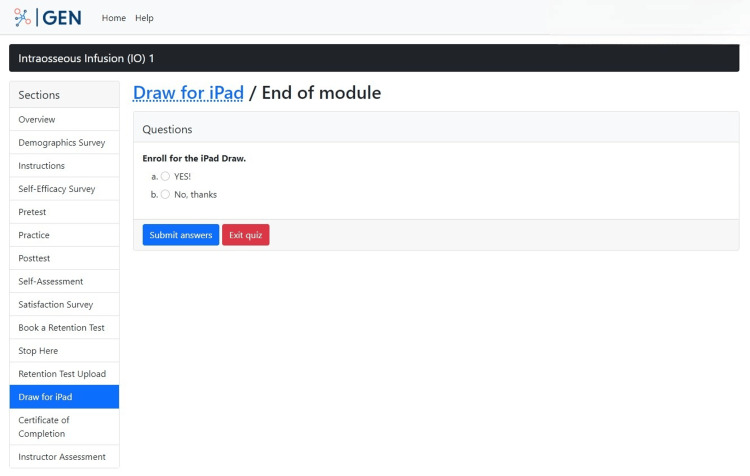
"Draw for an iPad" section in the GEN IO1 Module.

The "Draw for an iPad" section informs participants that they are entered into a draw to win an iPad after completing the study (Figure 18). This lets the participant know that they will be compensated for their time and effort in partaking in the study. The iPad was funded through the grants obtained for this study. 

**Figure 19:**
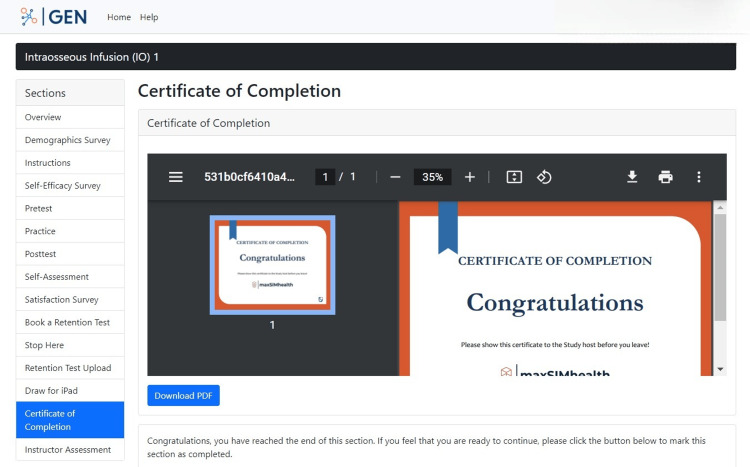
"Certificate of Completion" section in the GEN IO1 Module.

Finally, the last section entitled "Certificate of Completion" presents participants with a certificate that officially marks the end of the study (Figure 19). This allows the participants to understand that their contributions to the study are over and indicates to the researchers that the data for the participants have been fully collected. 

In sum, this GEN IO Module setup guides the participants through the study on their own and allows for the testing of a psychomotor educational model that works in conjunction with 3D-printed models. 

User Assessment 

**Figure 20:**
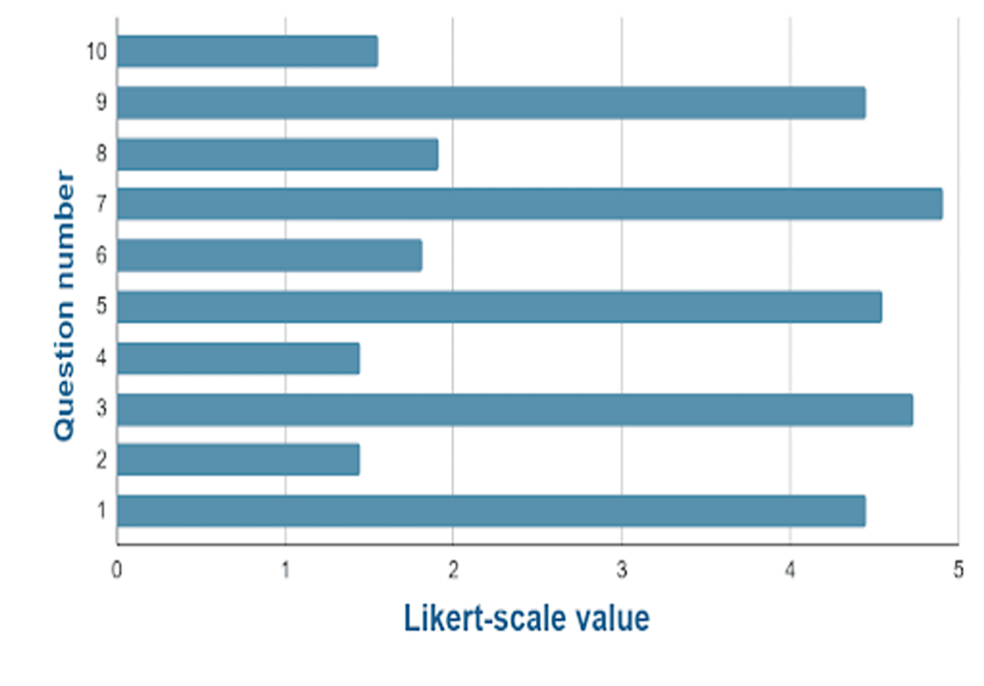
Responses of participants to 10 questions, relevant to the GEN IO Module, measured on a 5-point Likert scale translated into SUS scores. Likert scale: 1 = strongly disagree; 2 = disagree; 3 = neither agree nor disagree; 4 = agree; 5 = strongly agree. Q1: I think that I would like to use this product frequently. Q2: I found the product unnecessarily complex. Q3: I thought the product was easy to use. Q4: I think that I would need the support of a technical person to be able to use this product. Q5: I found the various functions in the product were well integrated. Q6: I thought there was too much inconsistency in the product. Q7: I imagine that most people would learn to use this product very quickly. Q8: I found the product very awkward to use. Q9: I felt very confident using the product. Q10: I needed to learn a lot of things before I could get going with this product. SUS: system usability survey; GEN IO: Gamified Educational Network Intraosseous

We received responses from 11 ACPs in total through the SUS questionnaire hosted on the GEN IO Modules. Their responses were presented as scores for GEN IO Modules 1 and 3. Six ACPS responded to their experience using the IO1 Module with the simple IO model. Five ACPs responded on their experience using the IO3 Module with the advanced IO model. The mean SUS score obtained in this study was 87.3, with a standard deviation of 7.6. Based on collected data, we can be 95% confident that the population SUS score is between 82.15 and 92.39 (margin of error of 5.12) and 97.5% confident that the population mean SUS score is above 82.15. The mean SUS score of 87.3 puts it on the 99% percentile rank, equivalent to an A+ grade based on the Sauro and Lewis grade scale [15]. Individual SUS scores for each question are shown in Table 3, and the data is also presented in Figure 20. 

**Table 3:** SUS questionnaire scores hosted on the GEN IO Modules. SD: standard deviation; SUS: system usability survey; GEN IO: Gamified Educational Network Intraosseous

Question	Likert scale frequencies						Mean	SD
	0	1	2	3	4	5		
1	-	-	-	1	4	6	4.45	0.69
2	-	8	2	-	1	-	1.45	0.93
3	-	-	-	-	3	8	4.73	0.47
4	-	8	2	-	1	-	1.45	0.93
5	-	-	-	-	5	6	4.55	0.52
6	-	5	2	1	1	-	1.82	0.98
7	-	-	-	-	1	10	4.91	0.30
8	-	6	2	1	2	-	1.91	1.22
9	-	-	-	1	4	6	4.45	0.69
10	-	7	3	-	-	-	1.55	0.93

Overall, the results indicate that a significant portion of the participants expressed that they would frequently use GEN IO. They found it to be not overly complex, user-friendly, and requiring minimal technical support and found the functions within GEN IO well integrated with little inconsistencies. Learning from GEN IO was perceived as a quick process, and using the program felt natural and not awkward. Participants conveyed a high level of confidence in their ability to use GEN IO, emphasizing that there wasn't much to be learned before using it. 

## Discussion

We created an LMS as described in this technical report for the simulation-based acquisition of psychomotor skills [10], pertaining to emergency IO access and infusion, that can be modified to structure training for the purpose of moving the training outside of the simulation laboratories (i.e., converting the training into a De-SBE model). A variety of studies have indicated that online learning has the potential to yield comparable benefits to in-person learning in the presence of a teacher [10,16-17]. 

Following the participants' utilization of GEN IO, the majority of the responses from the SUS questionnaire were positive given the average score of 87.3 with a standard deviation of 7.6. Most users of GEN IO thought it to be a great educational management tool characterized by its simplicity and ease of learning. The response to the question about participants' expectations on how quickly most people will pick up using GEN IO was quite favorable. This indicates that GEN IO is a simple LMS for acquiring valuable and practical medical procedures. The necessity to study a large amount of information prior to using the GEN IO was among the lowest mean scores, indicating that the participants found only a minimal amount of information necessary to grasp how to navigate the GEN IO. This underscores GEN's user-friendly design, emphasizing that effective use requires only minimal understanding of information. 

GEN IO, as a branch of De-SBE, serves as a cost-effective educational tool as it is provided free of charge to the learner. Given its pairing with affordably designed simulators, it stands in contrast to commercially available trainers. To provide an example, the Sawbones® (Seattle, USA) Intraosseous Access Injection Trainer is priced at $414 USD [18], while the production costs associated with our simple IO simulator and advanced IO simulator are $12.66 CAD and $53.89 CAD, respectively [11]. Clearly, the production of both our simple and advanced simulators can be achieved at significantly lower expenses relative to commercially accessible options [11]. The costs associated with development, such as designer hours and initial investments like the 3D printer and its related operational expenses (e.g., electricity, parts, maintenance), have not been factored into the production costs since they were previously funded through research and development grants unrelated to this project. The price of 3D printers can span hundreds to thousands of dollars [19]; however, public libraries offer alternatives where individuals can access 3D printing services without the requirement of owning a 3D printer themselves [20]. Understanding this, participants also have the option to utilize library services to print our digital design of the IO model which further demonstrates affordability. 

Nonetheless, concerns were raised by the participants. There was primarily a concern that was observed in-person during the testing with the participants. Problems with login came initially. A few individuals had trouble logging in using their Durham College email (the email that was to be used to log in to the GEN IO). The GEN IO technicians (AT and SA) were required to either manually assign an account during these times or offer a new password to the participant. The manual assignment of accounts results in delays, impacting the overall efficiency and smooth flow of the testing sessions. 

A key limitation was the file size constraint for uploading videos into GEN IO. Participants faced difficulties when attempting to upload videos into GEN IO sections that required a video to continue over to the next sections. Due to the large file size (>2.5 MB) of the videos, a few participants were unable to upload their videos to GEN IO. During this period, the videos were sent to the research assistants (AJ, DS, WF, MP), monitoring the testing with the participants, through email. The subsequent upload to the relevant GEN IO modules was managed by the GEN IO technicians (AT and SA). The video size uploads emerged as a recurring limitation, occasionally causing hassle for participants who were restricted from uploading videos exceeding the file size limit. It was occasionally inconvenient for these participants as it prolonged the time it took for them to submit their videos to their GEN IO profile and complete the overall GEN IO modules.

Based on these limitations, enhancements can be made. An area for potential improvement involves addressing technical issues, particularly the login problems that were identified. Manual assignment of participants to accounts was time-consuming which highlighted the need to prevent such login issues in the future for a seamless login process. Another time-consuming issue was the inability to upload videos into the GEN IO due to file size restrictions (>2.5 MB). To address this in the future, the GEN IO needs to be compatible with larger file sizes. Furthermore, the integration of more interactive features to promote greater engagement and social interaction among learners could be another improvement. This may include features such as discussion forums, live chat sessions with instructors or peers, and virtual group activities that allow learners to collaborate and foster solutions. To ensure the modules remain up-to-date and relevant, ongoing updates and revisions may be necessary to incorporate changes in clinical practice, technological advancements, and new research findings. Although this study did not have a Ce-SBE model to compare with the GEN IO (De-SBE), future research may focus on comparing the two models in further determining cost-effectiveness. As previously mentioned, a Ce-SBE model would encompass additional costs for time in compensating instructors, transportation costs for participants, and more [3]. De-SBE overcomes these constraints by providing flexibility to participants to engage in undisrupted practice remotely for as long as necessary until proficiency is achieved [3]. By incorporating these changes, the GEN IO can continue to provide learners with an effective and engaging educational experience that prepares them for success in their future practice.

As mentioned throughout this technical report, GEN IO also serves as an effective research tool because it provides us with the user experience data gathered from participants on their experiences in learning the IO access and infusion skill. Analyzing the participant data derived from the SUS scores provides us with an understanding of the GEN IO's effectiveness as an educational and research tool. 

Ultimately, based on the results received, GEN IO serves as a practical technological tool for a De-SBE model that supports competency-based training to provide participants with an independent and structured method of training and assists participants in preparation for traditional simulation (Ce-SBE).

## Conclusions

The GEN IO LMS described in this technical report is a user-friendly and viable solution for a De-SBE model to facilitate the training of paramedic students on the IO skill remotely. Through its adaptability, the system can be modified to fit the varying complexities of learning to match the diverse skill levels of participants. Its ability to provide clear instructions, monitor and collect participant performance and data, and allow integration of participant feedback demonstrates its great potential as a research and educational tool. If advanced with the appropriate resources and environment, the GEN IO can prove to be an effective tool in the acquisition of technical skills that can be accomplished in a remote setting.
